# The predictive value of bedside ultrasound to restore spontaneous circulation in patients with pulseless electrical activity: A systematic review and meta-analysis

**DOI:** 10.1371/journal.pone.0191636

**Published:** 2018-01-24

**Authors:** Chunshuang Wu, Zhongjun Zheng, Libing Jiang, Yuzhi Gao, Jiefeng Xu, Xiaohong Jin, Qijiang Chen, Mao Zhang

**Affiliations:** Department of Emergency Medicine, Second Affiliated Hospital, Zhejiang University School of Medicine Institute of Emergency Medicine, Zhejiang University, Hangzhou, China; University of Bern, University Hospital Bern, SWITZERLAND

## Abstract

**Background:**

The prognosis of pulseless electrical activity is dismal. However, it is still challengable to decide when to terminate or continue resuscitation efforts. The aim of this study was to determine whether the use of bedside ultrasound (US) could predict the restoration of spontaneous circulation (ROSC) in patients with pulseless electrical activity (PEA) through the identification of cardiac activity.

**Methods:**

This was a systematic review and meta-analysis of studies that used US to predict ROSC. A search of electronic databases (Cochrane Central, MEDLINE, EMBASE) was conducted up to June 2017, and the assessment of study quality was performed with the Newcastle-Ottawa Scale. Statistical analysis was performed with Review Manager 5.3 and Stata 12.

**Results:**

Eleven studies that enrolled a total of 777 PEA patients were included. A total of 230 patients experienced ROSC. Of these, 188 had sonographically identified cardiac activity (pseudo-PEA). A meta-analysis showed that PEA patients with cardiac activity on US were more likely to obtain ROSC compared to those with cardiac standstill: risk ratio (RR) = 4.35 (95% confidence interval [CI], 2.20–8.63; p<0,00001) with significant statistical heterogeneity (I^2^ = 60%). Subgroup analyses were conducted: US evaluation using only on the subxiphoid view: RR = 1.99 (95% CI, 0.79–5.02; p = 0.15); evaluation using various views: RR = 4.09 (95% CI,2.70–6.02; p<0.00001).

**Conclusions:**

In cardiac arrest patients who present with PEA, bedside US has an important role in predicting ROSC. The presence of cardiac activity in PEA patients may encourage more aggressive resuscitation.

## Introduction

Cardiac arrest (CA) is an urgent and fatal condition frequently encountered in the emergency room, with 110.8 cases per 100,000 person-years experiencing emergency medical service (EMS) system-assessed out-of-hospital cardiac arrests (OHCA) in the United States. Despite some improvements in the performance of cardiopulmonary resuscitation (CPR) over the past decades, the survival-to-hospital-discharge rate is only 10.6% [1]. Pulseless electrical activity (PEA) is a particular type of CA in which patients have organized electrical activity without a palpable pulse. Over the past two decades, the incidence of PEA has increased to 35%-40% of all CAs [2]. While the patients with PEA has a poorer prognosis (survival rate: 2.4%), compared to those with a shockable rhythm (40%) [3]. PEA can be sub-divided into pseudo-PEA and true-PEA according to the presence or absence of cardiac activity on ultrasound (US). The survival rate of pseudo-PEA has been reported to be significantly higher than that of true-PEA.

Most studies that have assessed the value of bedside US in predicting survival in PEA patients are limited in terms of their small sample size and discrepancies between studies. Two previous meta-analysises evaluated the value of focused echocardiography in predicting outcome of resuscitation in patients with CA, but no specially targeted at PEA patients [4, 5]. Therefore, we conducted a systematic review and meta-analysis to determine the accuracy of US for predicting the restoration of spontaneous circulation (ROSC) in PEA patients.

## Methods

The present meta-analysis was conducted and reported according to the preferred reporting items for systematic reviews and meta-analyses (PRISMA) (S1 Checklist) [6].

### Data sources

An extensive search of the MEDLINE, EMBASE, and Cochrane library databases from inception to June 2017 was conducted. The following medical subject headings (MESH) or text words were used: (1) “pulseless electrical activity” or “electrical mechanical dissociation”; (2) “cardiopulmonary resuscitation” [Mesh] or “heart arrest” [Mesh]; and (3) “ultrasonography” [Mesh]. The three subsets were combined using the Boolean term “AND” to obtain a subset of citations relevant to our research question. The search strategy was adjusted to account for differences in indexing between the databases (S1 File). To identify additional studies, the reference lists of each of the selected articles were also searched.

### Eligibility criteria

Studies were included if the following criteria were met: (1) adult patients with PEA were included in the study; (2) cardiac US was performed during resuscitation to assess the presence or absence of cardiac activity; (3) the restoration of spontaneous circulation (ROSC) was defined as the primary outcome; (4) the papers were prospective, observational studies; (5) were written in Enhlish; and (6) a 2×2 contingency table could be constructed from obtained data. Two reviewers (C.S.W. and Z.J.Z.) independently evaluated studies for inclusion in the systematic review. Any discrepancies in paper selection and data extraction were resolved by consensus. A third reviewer (L.B.J.) was consulted in the event of any unresolved issues.

### Quality assessment

Assessments of quality and bias risk were independently performed by two reviewers (J.F.X. and Y.Z.G.) using the Newcastle-Ottawa Scale (NOS) [7]. This tool consists of eight items in four categories, including crowd selection, comparability, exposure assessment, and outcome evaluation. It evaluates the quality of a study using a semi-quantification principle star system, in which the maximum is nine stars. A maximum of two stars can be given for comparability. The studies with the NOS stars greater than 7 would be considered to be high quality. Any disagreements were resolved by consensus.

### Data extraction

The following items were extracted independently by two reviewers (C.S.W. and Z.J.Z.) using a predetermined sheet: study setting, study location, study scene, mean age, sample size, patient characteristics, US views, US operators’ experience, and the time that US was performed. The number of patients who suffered PEA with or without cardiac contractions, and the number of patients who did or did not experience ROSC among those with or without cardiac contractions were obtained from the studies and were used to construct a 2×2 contingency table.

### Statistical analysis

Statistical analysis was performed with Review Manager 5.3 (The Nordic Cochrane Centre, The Cochrane Collaboration, Copenhagen, Denmark) and Stata 12 (Stata Statistical Software: Release 12: Stata CorpLP, College Station, TX, USA) [8]. Data from primary studies were summarized in a 2×2 table of test results, and the association of cardiac activity with CA outcome was estimated for each study by a risk ratio (RR) along with its 95% confidence interval (CI). All pooled outcome measures were determined using random-effects model as described by DerSimonian and Laird [9]. Heterogeneity was assessed by I^2^ statistics [10]. Statistical significance was assigned when p value was <0.05. In addition, a meta-regression analysis was conducted to screen the factors resulting in heterogeneity. This analysis explores whether the association exists between possible variables (study setting, study location, study scene, sample size, patient characteristics, and US views) and ROSC, along with the direction of that association. If one or more affected variables of heterogeneity were screened, subgroup analysis based on the hypothesis was conducted to control the heterogeneity. Then, we deleted one study from the overall pooled analysis each time to check the effect of the altered data set on the overall RRs.

In addition, a Begg’s text was used to assess the publication bias. A value of “Pr > |z|” less than 0.05 indicated potential publication bias.

## Results

### Study selection and characteristics

**Fig 1:**
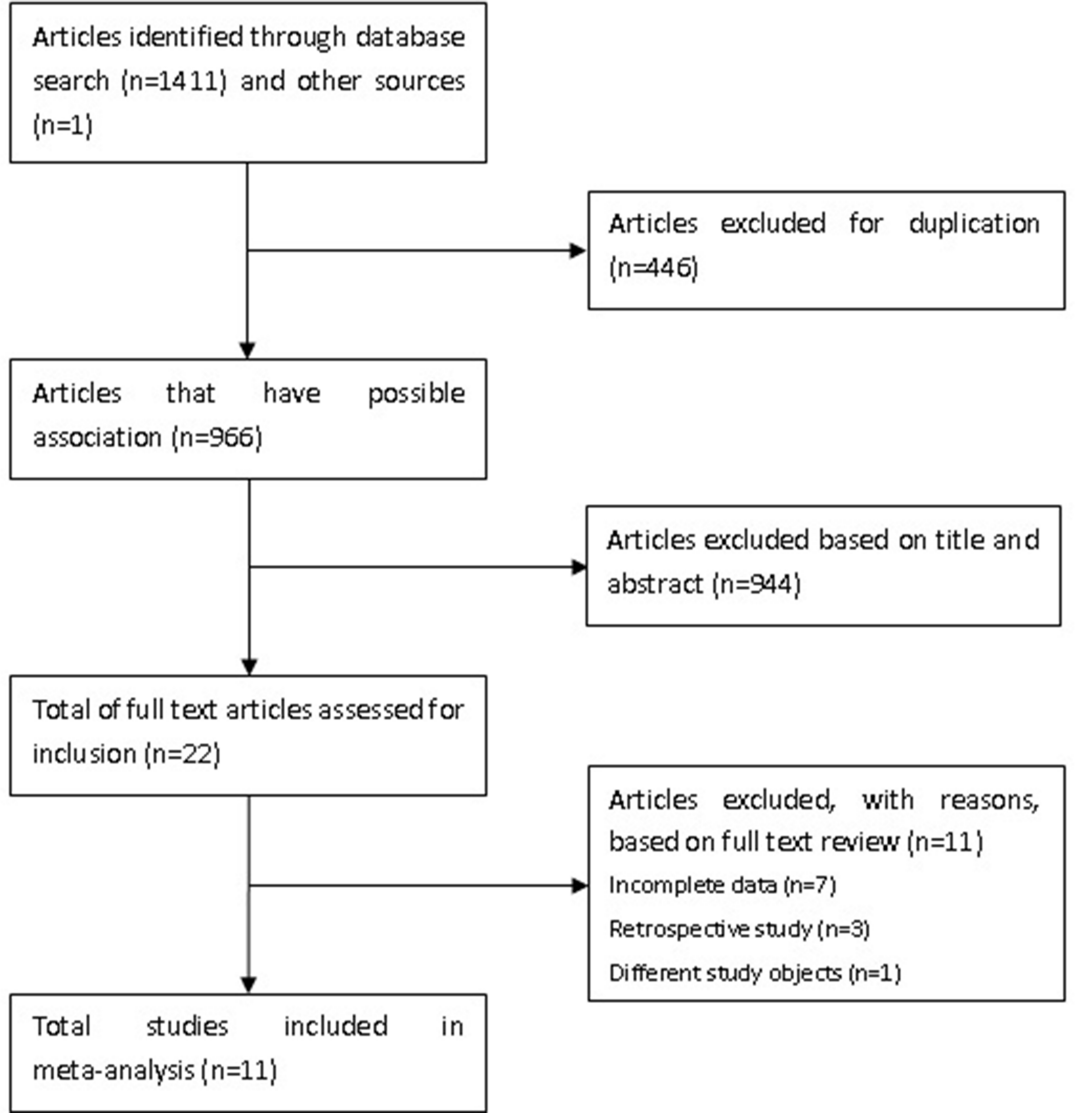
The flowchart of the selection process.

A flow diagram of the study selection protocol is shown in Fig 1. A total of 1412 articles were identified, of which 446 duplicated studies were excluded. After reviewing the titles and abstracts, a further 944 articles were excluded. The full manuscripts of 22 articles were reviewed in detail and 11 of them were excluded (seven studies had incomplete data, three were retrospective studies, and one had a different objective from our study). There was very good agreement for abstracts (Cohen’s kappa 0.92) and full agreement (Cohen’s kappa 1.00) at the full article stage. Finally, 11 studies with 777 patients were eligible for the meta-analysis [11–21].

The characteristics of the included 11 studies are shown in Table 1. Seven of the eligible studies’ objects were non-trauma patients [11, 12, 16–20], and four studies enrolled both trauma and non-trauma patients [13–15, 21]. The US evaluations in three studies were initiated out of hospital [11, 13, 17], while other studies’ evaluations were performed in-hospital [12, 14–16, 18–21]. The US examinations were performed using various cardiac windows, including the subcostal, apical, and parasternal four chamber views, but there were three studies in which US images were acquired only in the subxiphoid view [11, 14, 21].

**Table 1:** Characteristics of the 11 studies included in a systematic review and diagnostic accuracy meta-analysis.

Study ID	Study setting	Study location	Study scene	Mean age (years)	Sample size (n)	Patients’ characteristics	US views	US operators’ experience	The time US was performed
Aichinger 2012	Unknown	Austria	Pre-hospital	70.3	11	Non-traumatic	Subxiphoid	A 2-h course in echocardiography	During a rhythm and pulse check
Blaivas 2001	Single	USA	In-hospital	71	38	Non-traumatic	Subxiphoid; if unable to obtain, use parasternal views	Ultrasound trained and credentialed	The duration of the pulse check
Breitkreutz 2010	Single	Germany	Pre-hospital	65±15	51	Both	One of three views	Having undergone the standard FEER training program	During an ALS-conformed interruption of CPR
Flato 2015	Single	Brazil	In-hospital	59.75±18.11	32	Both	Various views	Had a 60-min lecture on ALS-conformed-TTE	During the intervals for rhythm check for 10s
Chardoli 2012	Multicenter	Iran	In-hospital	58±6.1	50	Both	Subxiphoid	Attended a teaching course to performing echocardiography	Just in the first NFI
Salen 2001	Multicenter	USA	In-hospital	Unknown	55	Non-traumatic	Subxiphoid view; the apical view as an adjunct in obese patients	Received a 4-h trauma sonography course	During the pulse check pause of the ALS
Salen 2005	Multicenter	USA	Pre-hospital/in-hospital	16–94	34	Non-traumatic	Subxiphoid or parasternal	Physician sonographers	Examinations during the pulse check
Tayal 2003	Single	USA	In-hospital	57±15	20	Non-traumatic	Various views	Trained with a 20-h ultrasound course	During CPR
Gaspari 2016	Multicenter	USA and Canada	In-hospital	64.2±17.4	414	Non-traumatic	Subxyphoid or parasternal long axis views	Emergency physician’s credentialed in bedside ultrasound by their individual hospitals	During pauses in resuscitation
Kim 2016	Single	Korea	Pre-hospital	63.9±14.5	8	Non-traumatic	Subcostal or parasternal window	The senior emergency resident or emergency specialist who had≥3 years’experience in emergency echocardiography	During pulse checks
Tomruk 2012	Single	Turkey	In-hospital	61.6±17.9	64	Both	Subxiphoid cardiac approach	Theoretical and hands-on training in cardiac ultrasonography	During the initial assessment

PEA, pulseless electrical activity; ROSC, restoration of spontaneous circulation which was defined as a return of spontaneous circulation for ≥20 mins or ROSC upon hospital admission; US, ultrasound; ALS, advanced life support; NFI, no flow interval; TTE, transthoracic echocardiography; in-hospital indicates that bedside ultrasound was not used until arrival at hospital; pre-hospital indicates that ultrasound was used at the scene; Unknown, the data was unable to be obtained; FEER, focused echocardiographic evaluation in resuscitation; CPR, cardiopulmonary resuscitation.

### Quality assessment

Overviews of the quality assessments of the 11 studies are shown in Table 2.

**Table 2:** Assessment of the quality of the eleven studies.

	Aichinger 2012(9)	Blaivas 2001(10)	Breitkreutz 2010(11)	Flato 2015(13)	Chardoli 2012(12)	Salen 2001(16)	Salen 2005(4)	Tayal 2003(17)	Gaspari 2016(14)	Kim 2016(15)	Tomruk 2012(18)
Representativeness of the exposed cohort	*	*	*	*	*	*	*	*	*	*	*
Selection of the nonexposed cohort	*	*	*	*	*	*	*	*	*	*	*
Ascertainment of exposure	*	*	*	*	*	*	*	*	*	*	*
Demonstration that outcome of interest was not present at start of study	*	*	*	*	*	*	*	*	*	*	*
Comparability of cohorts on the basis of the design or analysis	**	-	-	**	-	-	-	-	**	**	**
Assessement of outcome	*	*	*	*	*	*	*	*	*	*	*
Was follow-up long enough for outcomes to occur	*	*	*	*	*	*	*	*	*	*	*
Adequacy of follow up of cohort	*	*	*	*	*	*	*	*	*	*	*

*: One star

**: Two stars.

### Meta-analysis

**Fig 2:**
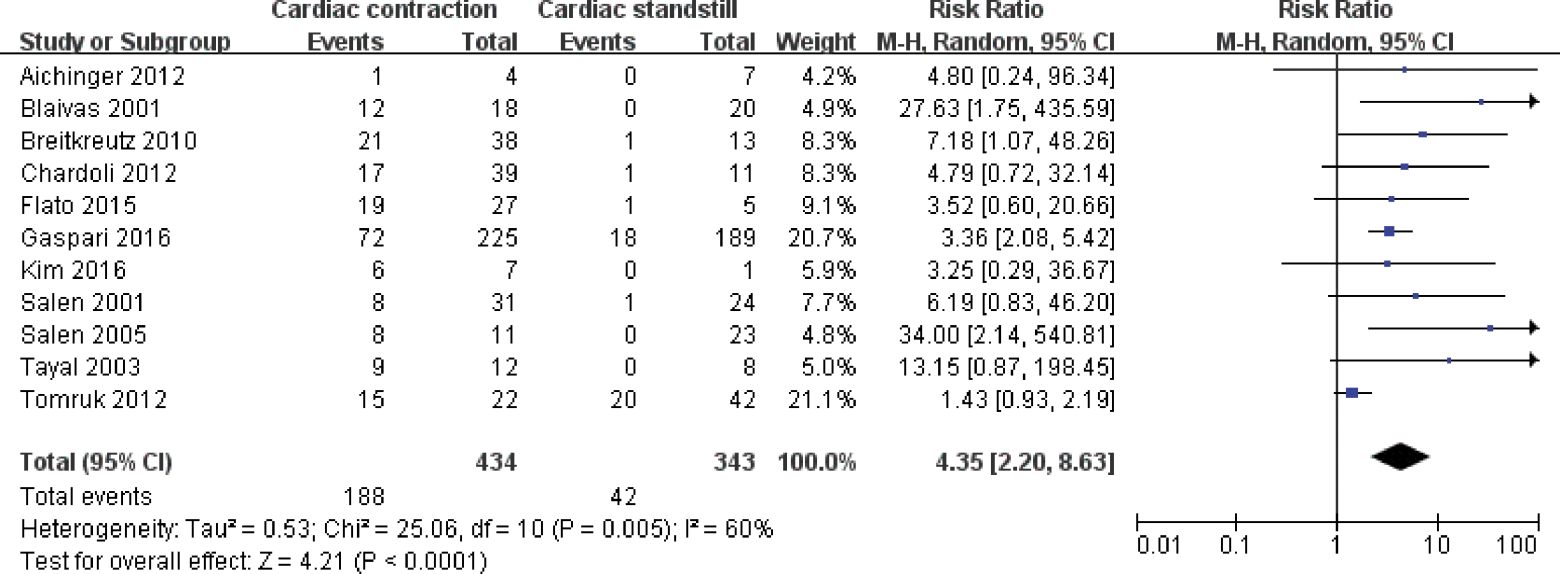
Forest plot of the pooling effects of cardiac activity on the restoration of spontaneous circulation. Pulseless electrical activity (PEA) can be sub-divided into pseudo-PEA and true-PEA according to the cardiac contraction or cardiac standstill on ultrasound (US).

Eleven studies involving 777 subjects met the defined inclusion criteria. Forty-two of 343 true-PEA patients obtained ROSC, and 188 of 434 pseudo-PEA patients obtained ROSC. Not all of the studies provided information on hospital discharge, but the 15 patients reported to have survived to hospital discharge were all pseudo-PEA. A random-effects model was applied to (pooled RR 4.35, 95% CI: 2.20–8.63; p<0.00001) for the high level of statistival heterogeneity (I^2^ = 60%). Therefore, a PEA patient with cardiac activity was 4.35 times more likely to experience ROSC than one with cardiac standstill (Fig 2).

### Meta-regression analysis

**Fig 3:**
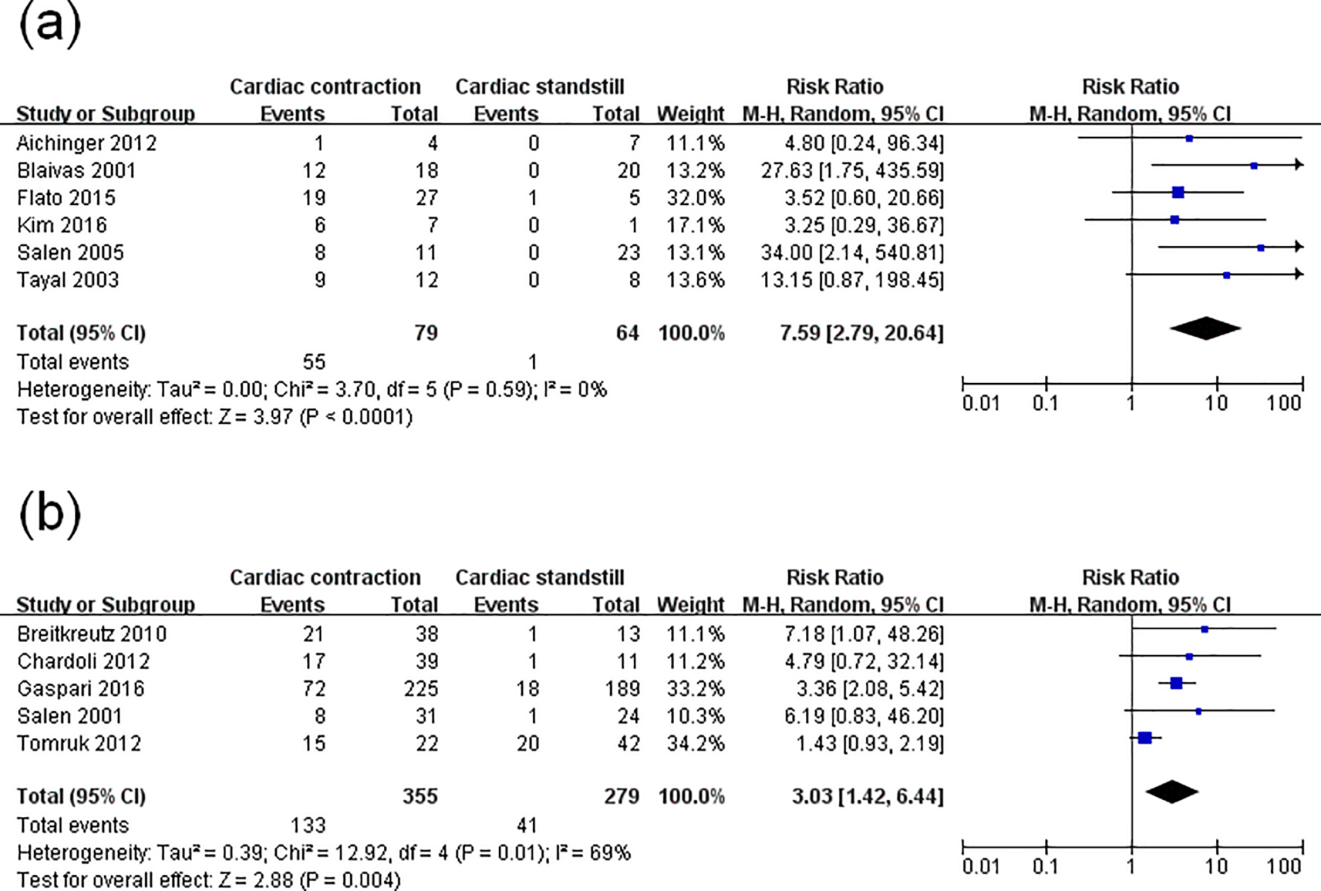
Forest plot of the subgroup analysis of cardiac activity on the restoration of spontaneous circulation. (**a**) studies whose sample size was n<50; (**b**) studies whose sample size was n≥50.

**Fig 4:**
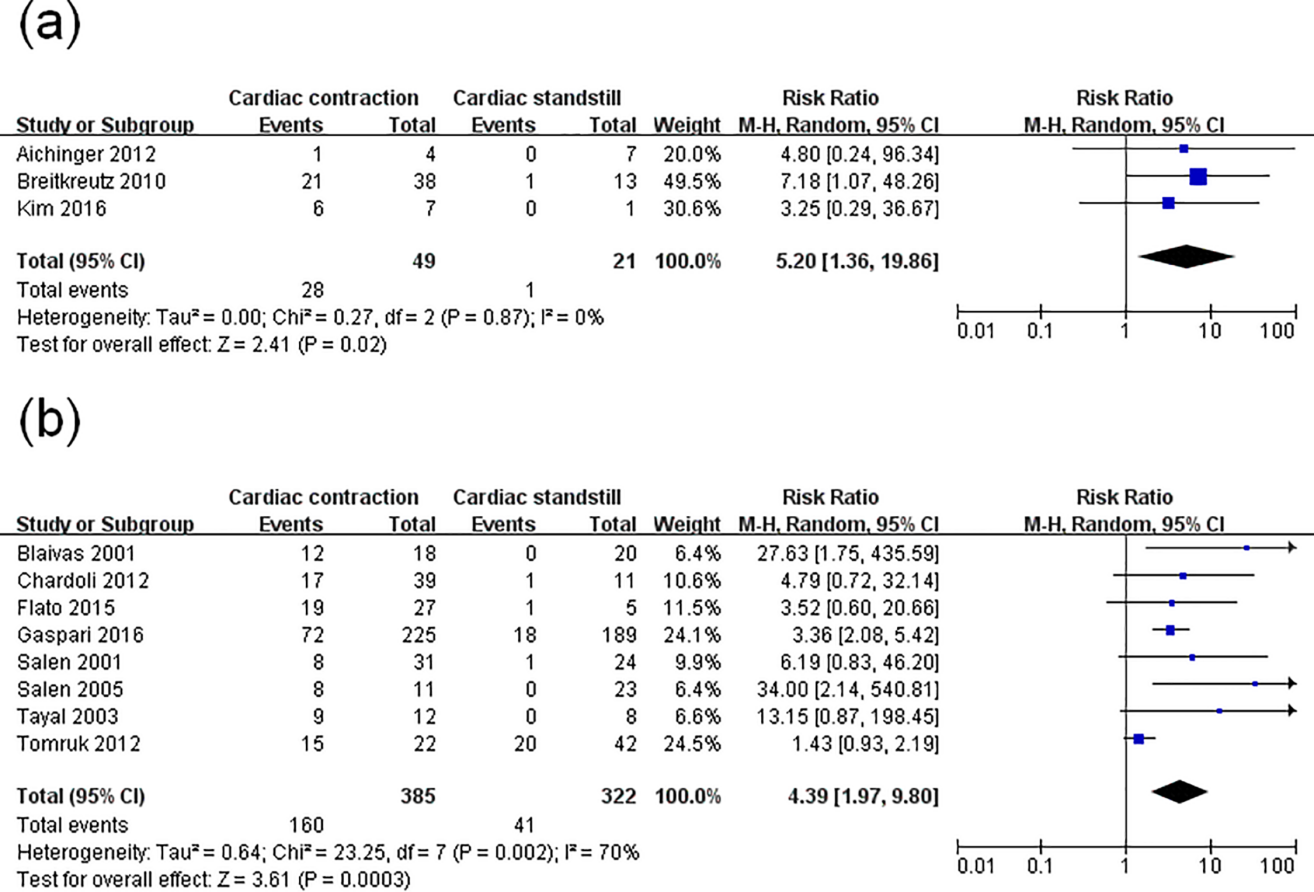
Forest plot of the subgroup analysis of cardiac activity on the restoration of spontaneous circulation. (**a**) studies whose ultrasound evaluation occurred pre-hospital; (**b**) studies whose ultrasound evaluation occurred in-hospital.

Since obvious heterogeneity was observed, a meta-regression analysis was performed to assess the specific variables (study setting, study location, study scene, patients’ characteristics, sample size, US views) concerned. Sample size (p = 0.003) and study scene (p = 0.002) were found to be major factors associated with ROSC. Then, a subgroup analysis based on these two factors was conducted. In one subgroup analysis (with sample size n<50 and n≥50), the pooled RR was 7.59 (95% CI, 2.79–20.64; p<0.00001) with no evidence of statistical heterogeneity (I^2^ = 0%), and 3.03 (95% CI, 1.42–6.44; p = 0.004) with evidence of significant statistical heterogeneity (I^2^ = 69%) (Fig 3). In another subgroup analysis (with the US examination performed pre-hospital and in-hospital), the RR was 5.20 (95% CI, 1.36–19.86; p = 0.02) without statistical heterogeneity (I^2^ = 0%) and 4.39 (95% CI, 1.97–9.80; p = 0.0003) with significant statistical heterogeneity (I^2^ = 70%) (Fig 4). Thus, the two factors could not completely explain the heterogeneity. This may be attributed to other factors such as the difference of pathogenesis, downtime, time down without CPR, CPR duration, or medications used, which were not analyzed here. Moreover, the instability of the regression model caused by the limited number of included studies was also a key factor.

**Fig 5:**
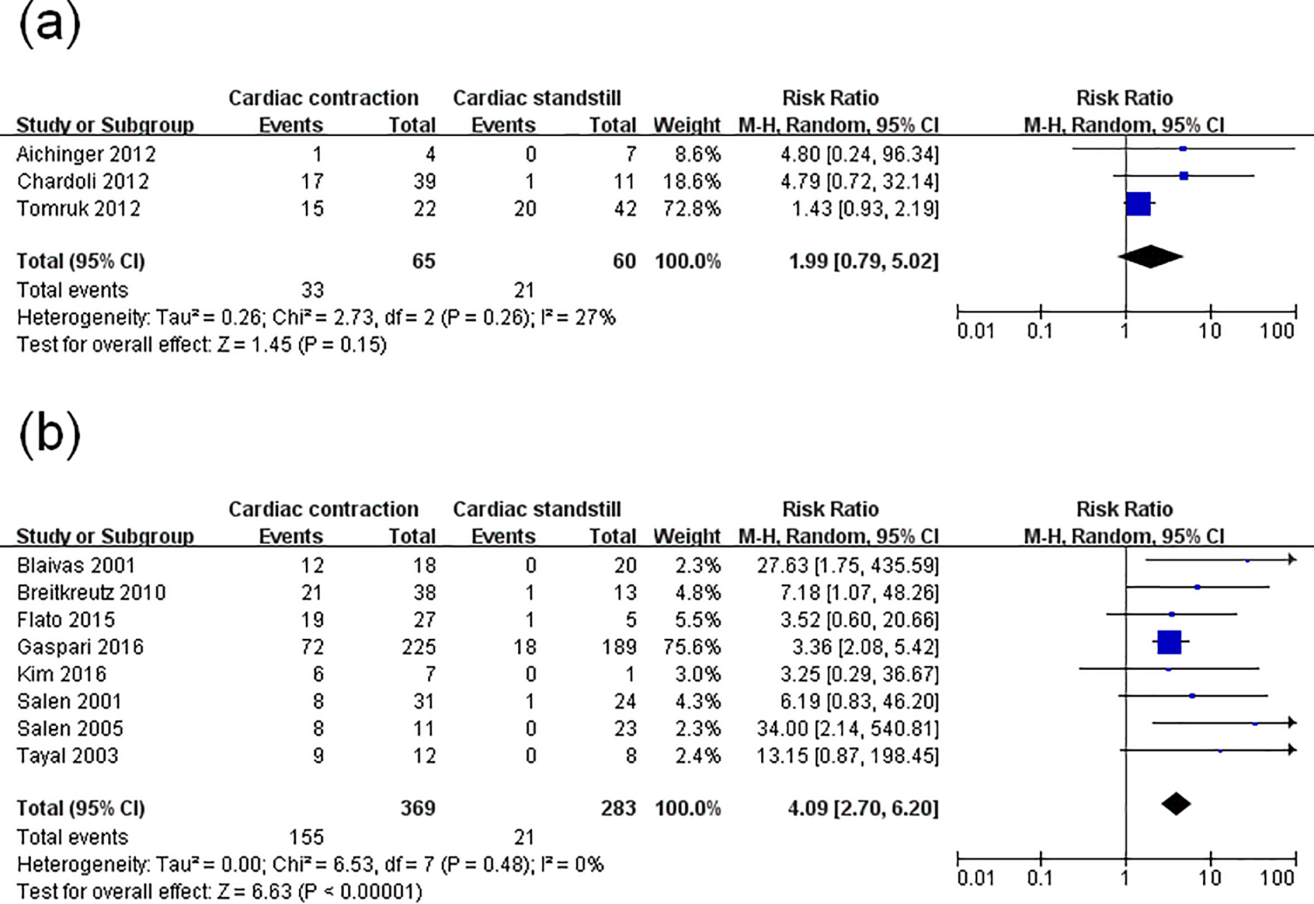
Forest plot of the subgroup analysis of cardiac activity on the restoration of spontaneous circulation. (**a**) studies whose ultrasound evaluation focused only on the subxiphoid; (**b**) studies whose ultrasound evaluation used various views.

Subsequently, another subgroup analysis was conducted to trace the origin of the heterogeneity. Among the 11 studies, the US images in three studies were obtained using the subxiphoid view only, while the other studies’ US evaluations were performed using various views. In the group using subxiphoid view, no significant difference between pseudo-PEA and true-PEA in the rate of ROSC (pooled RR 1.99, 95% CI: 0.79–5.02; p = 0.15). There was a low level of statistical heterogeneity (I^2^ = 27%). The RR of the other group which using various views was 4.09 (95% CI, 2.70–6.20; p<0.00001) with no heterogeneity (I^2^ = 0%) (Fig 5).

### Sensitivity analysis

Only one study (Tomruk 2012) [21] significantly changed the between-study heterogeneities. After the deletion of this study, the heterogeneity vanished while the association remained significant (RR, 4.13; 95% CI: 2.76–6.18; p<0.00001) without statistical bias (I^2^ = 0%).

Begg’s test was completed to assess publication bias, and the result yielded a score of Pr>|z| = 0.533, indicating no publication bias.

## Discussion

The present systematic review and meta-analysis has shed important light on the accuracy of bedside US as a prognostic tool during CPR. The results showed that the pool RR of the US predicting ROSC was 4.35 (95% CI, 2.20–8.63; p<0.00001). Therefore, bedside US may be a fairly effective (although not definitive) test for predicting ROSC, and may assist doctors in determining when to terminate or optimize CPR.

Cardiac arrest remains the main cause of death despite improvements to the cardiopulmonary resuscitation technique. The CPR procedure entails much time and medical resources, but the prognosis is dismal. One of the dilemma is in determining when to cease CPR or continue with optimal resuscitation. Downtime, bystander CPR, initial arrest rhythm, duration of resuscitative efforts, and age are widely suggested to be important prognostic parameters. However, they are not fully reliable, and the decision is usually affected by personal biases. Thus, an accurate, time-efficient method of predicting ROSC in the emergency department is required. The ultrasound scanning can be performed during the pulse check, and does not interfere with chest compression. Recently some studies have described the prognostic ability of bedside US [11–13, 15, 16, 19, 22–24]. Two previous meta-analysises suggested that the absence of cardiac activity on ultrasound could reliably predict failure of CPR, and guide the termination of resuscitation efforts in CA patients. However, the value of US during CPR is still limited [4, 5].

Cardiac arrest victims who present with PEA have accounted for up to 30% of cases during the past two decades [25]. The survival rate of PEA is even lower (2.4%), while the decision as to whether to stop CPR is more difficult due to the remaining electrical activity of PEA, compared to those with a shockable rhythm. Pseudo-PEA is amenable to simple intervention and has a higher survival rate than true-PEA. The use of US to judge cardiac activity may have a greater predictive value in PEA patients rather than in all CA patients. The objective of this review is to summarize current evidence on the predicitive value of US for the ROSC in PEA patients.

Compared with the previous meta-analysises by Blyth [4] and Tsou [5], which included all CA patients, this is the first study to assess the summary value of US in PEA patients individually. In this study, 230 of 777 PEA patients obtained ROSC. Of these, only 42 had true-PEA but none survived to hospital discharge. The identification of true-PEA on ultrasound could predict the failure of resuscitation and guide the termination of CPR. On the subgroup analysis, the RR of the studies performed using various views was significately, indicating a PEA patient with cardiac activity identified by ultrasound scanning in various views was 4.09 times more likely to experience ROSC than one with cardiac standstill. Ultrasound scanning by single subcostal view failed to identify any clear improvement of ROSC in pseudo-PEA by ultrasound scanning. This may be because the number of studies published were too low to be statisical significance. Moreover, the heterogeneity of studies (I^2^ = 27%) may be also a key factor. However, the trend was still a increased rate of ROSC in pseudo-PEA patients. On the sensitivity analysis, it is noteworthy that the deletion of Tomruk’ study, the heterogeneity reduced significantly. This may be also explained by the inaccuracy due to the use of single subcostal view.

Although, the systematic review and meta-analysis increased the sample size, the inferences estimated from the pooled data are subject to the limitations of the primary studies. The majority of the included studies have methodlogically shortcoming in sample size, design, outcome measures, and statistical analysis. In addition, despite the fact that the physicians had been trained by US-certified physicians, it is possible that the same degree of very minimal motion could be described as “no motion” by one clinician, and “positive motion” or “a twitch of motion” by another. Even though it is claimed, academically, that the US evaluation does not interrupt chest compression, this is not always true in a clinical setting. The additional step of US evaluation inevitably affects the ACLS process, especially when performed by unskilled operators. Additionally, the US evaluation would disturb the application of mechanical chest compressions. The reference standard of all studies is ROSC, but the definition of ROSC outcome is not consistent among different studies. For example, the ROSC was defined as the return of s spontaneous pulse in Schuster’s [24] study; in Chardoli’s [14]study, ROSC meant the presence of a palpable pulse and detectable blood pressure for at least 10 seconds; while in Flato’s [15] study, the ROSC needed to remain more than 20min. Although CPR was continued, regardless of the US findings in all studies, the duration was not fixed. In most studies, the US findings were not blinded to CPR providers. These are major biases in the included studies in this research. Although it is still insufficient to support using bedside US in isolation to decide whether to continue resuscitation efforts. The presence or absence of cardiac contraction can provide physicians with further information to assist the difficult decision-making process associated with whether to terminate CPR [4]. Further multicentric and high-quality studies are required in the future to provide further, confirmed evidence and guidelines for physicians.

There are several limitations to this study. Due to the few published studies and low occurrence of PEA, the pooled sample size is still relatively small, which leads to a relatively wide confidence interval. Besides, we may also have missed some studies published in non-English. However, the lower confidence interval of the pooled RR was 2.20 in our study, which indicated the predictive value of bedside US in PEA patients’ ROSC. In addition, the majority of the included studies used a convenient, non-consecutive sample because of several methodological and practical constraints. Moreover, there are other factors such as time down without CPR, time to EMS’s arrival, length of CPR and the underlying health of the patients that are related to the prognosis of CA [26]. Since the relevant data cannot be acquired integrally, correlation analysis regarding these factors was not performed. Although a meta-regression and subgroup analysis were conducted to trace the heterogeneity, and Begg’s test was completed to observe publication bias, the small sample size and limited number of included studies affected the tests’ efficiency.

## Conclusions

In CA patients who present with PEA, bedside US has an important value in predicting ROSC. The presence of cardiac activity in PEA patients may encourage more aggressive resuscitation. Alternatively, the absence of cardiac activity under US could be promoted as a way of confirming a poor prognosis and used to support the decision to terminate resuscitative efforts.

## Supporting information

S1 Checklist PRISMA checklist. (DOC)

S1 File Full search strategies in Medline, EMBASE, and COCHRANE. (DOC)
