# Supplementary material for: The predictive value of bedside ultrasound to restore spontaneous circulation in patients with pulseless electrical activity: A systematic review and meta-analysis
Source: PLoS One. 2018 Jan 24;13(1):e0191636. doi: 10.1371/journal.pone.0191636 (PMC5783414; doi:10.1371/journal.pone.0191636)
Supplement: S1 File — (DOC) [file pone.0191636.s002.doc]

**ELECTRONIC SUPPLEMENT**

**Search strategy----Medline search**

1. PEA
2. pulseless electrical activity
3. EMD
4. electrical mechanical dissociation
5. electromechanical dissociation
6. cardiac contraction
7. cardiac activity
8. cardiac movement
9. 1 OR 2 OR 3 OR 4 OR 5 OR 6 OR 7 OR 8
10. CPR
11. Cardiopulmonary resuscitation [Mesh]
12. resuscitation
13. heart arrest [Mesh]
14. cardiac arrest
15. ACLS
16. advanced cardiac life support
17. sudden death
18. sudden cardiac death
19. 10 OR 11 OR 12 OR 13 OR 14 OR 15 OR 16 OR 17 OR 18
20. ultrasonography [Mesh]
21. bedside ultrasound
22. sonography
23. echocardiography [Mesh]
24. cardiac sonography
25. transthoracic echocardiography
26. TTE
27. 20 OR 21 OR 22 OR 23 OR 24 OR 25 OR 26
28. 9 AND 19 AND 27

**Search strategy----EMBASE search**

1. ‘pea’/exp OR ‘pea’ OR ‘pulseless electrical activity’ OR ‘emd’ OR ‘electrical mechanical dissociation’ OR ‘cardiac contraction’/exp OR ‘cardiac contraction’ OR ‘cardiac activity’ OR ‘cardiac movement’/exp OR ‘cardiac movement’
2. Ultrasonography:ab,ti OR bedside ultrasound:ab,ti OR sonography:ab,ti OR echocardiography:ab,ti
3. ‘cpr’:ab,ti OR ‘cardiopolmonary resuscitation’:ab,ti OR ‘heart arrest’:ab,ti OR ‘cardiac arrest’:ab,ti OR ‘acls’:ab,ti OR ‘advanced cardiac life support’:ab,ti OR ‘sudden death’:ab,ti
4. 1 AND 2 AND 3

**Search strategy----COCHRANE search**

1. “pulseless electrical activity” or electrical mechanical dissociation or cardiac activity or cardiac contraction or cardiac movement in Trial (Word variations have been searched)
2. “heart arrest”:ti,ab,kw or “cardiac arrest”:ti,ab,kw or “cardiopulmonary resuscitation”:ti,ab,kw or Advanced Cardiac Life Support:ti,ab,kw (Word variations have been searched)
3. “ultrasonography”:ti,ab,kw or “bedside ultrasound”:ti,ab,kw or “echocardiography”:ti,ab,kw or “sonography”:ti,ab,kw (Word variations have been searched)
4. 1 and 2 and 3
